# Distinct cervical microbiome and metabolite profiles before and after menopause: implications for cervical cancer progression

**DOI:** 10.3389/fcimb.2025.1589277

**Published:** 2025-07-16

**Authors:** Rie Kawasaki, Iwao Kukimoto, Eiji Nishio, Sayaka Otani, Haruki Nishizawa, Yasuhiro Maeda, Aya Iwata, Takuma Fujii

**Affiliations:** ^1^ Department of Gynecology, Fujita Health University, School of Medicine, Toyoake, Aichi, Japan; ^2^ Department of Obstetrics and Gynecology, Fujita Health University, School of Medicine, Toyoake, Aichi, Japan; ^3^ Pathogen Genomics Center, National Institute of Infectious Diseases, Musashi-murayama, Tokyo, Japan; ^4^ Open Facility Center, Fujita Health University, Toyoake, Aichi, Japan; ^5^ Fujita Health University Okazaki Medical Center, Okazaki, Aichi, Japan

**Keywords:** cervical cancer, cervical intraepithelial neoplasia, microbiome, metabolites, microRNA, cytokines, menopause

## Abstract

**Introduction:**

Cervical cancer is the fourth most common malignancy in women and is primarily caused by persistent infection with high-risk human papillomavirus (HPV). In addition, host immune responses, genetic factors, and lifestyle habits also have etiological roles. The cervicovaginal microbiome undergoes dynamic changes during menopause, which may be involved in the progression of cervical neoplasia. We aimed to elucidate the association between cervical microenvironmental changes and the progression of cervical neoplasia before and after menopause by integrating analyses of the cervical microbiome, related metabolites, cytokines, and microRNAs.

**Methods:**

A total of 248 HPV-positive women with cervical neoplasia, including 17 with cervical intraepithelial neoplasia (CIN1), 80 with CIN2, 82 with CIN3, and 69 with squamous cell carcinoma (SCC), were enrolled. As normal controls, 48 HPV-negative healthy women were included. Each group was stratified based on the mean menopausal age of 50 years. Cervical mucus was analyzed according to the methods outlined below. The microbiota was profiled by 16S rRNA gene sequencing, metabolites were analyzed by ultra-HPLC-tandem mass spectrometry, RT-qPCR was used for miRNA expression analysis, and RANTES levels were quantified by multiplex bead array. Data analysis was performed using MicrobiomeAnalyst and MetaboAnalyst.

**Results:**

In the SCC group, *Prevotella* and *Atopobium* were the key bacterial genera among the younger group, while *Peptoniphilus*, *Fusobacterium*, and *Porphyromonas* were more prevalent in elderly group (LDA score > 4.5). We observed a consistent positive correlation between *Atopobium* and xanthine in younger groups with CIN2 or worse (*p* < 0.0001). However, no such correlations were detected in elderly women. In addition, *Atopobium*, *Adlercreutzia*, and *Gardnerella* showed significant positive correlation with nicotinic acid in younger women with SCC compared to the elderly women (*p* < 0.0001). In the younger SCC women, several metabolites were significantly elevated in groups with high expression levels of RANTES, miR-20b-5p, and miR-155-5p.

**Conclusion:**

The cervical microbiome undergoes changes during menopause, and may influence disease progression by interacting with metabolites, cytokines, and miRNAs. These results highlight the potential for personalized medicine for cervical cancer that is tailored to different age groups.

## 1 Introduction

Cervical cancer is one of the leading causes of cancer-related morbidity and mortality among women worldwide and ranks fourth in terms of incidence. Each year, approximately 660,000 new cases of cervical cancer are diagnosed, and 350,000 deaths are recorded globally (Bray et al., 2024). The primary cause of cervical cancer is persistent infection with high-risk human papillomavirus (HPV) types (Bosch et al., 2002; Wang et al., 2018). However, not all HPV infections lead to malignancy, and the mechanisms regulating this progression remain poorly understood (Lehoux et al., 2009; Bowden et al., 2023; Guo et al., 2023).

Host factors such as immune responses, genetic predisposition, and the cervical microenvironment also play critical roles in cervical carcinogenesis (George et al., 2023; Yousaf et al., 2024; Zhao et al., 2024; Castro-Muñoz et al., 2022; Jia et al., 2023; Vallejo-Ruiz et al., 2024). Additionally, persistent HPV infections and hormonal influences are known to increase the risk of progression to cervical intraepithelial neoplasia (CIN) and cervical cancer (Roura et al., 2016; Bowden et al., 2023; Luvián-Morales et al., 2024; Na et al., 2023; Letafati et al., 2024). These findings highlight the complexity of cervical cancer development and underscore the need for comprehensive analyses. Recent studies suggest that the tissue microbiome significantly impacts the tumor microenvironment (Constantin et al., 2024; Fong Amaris et al., 2024; Benešová et al., 2023). An imbalance in the vaginal and cervical microbiota can increase the risk of bacterial vaginosis (BV) and HPV infection, and that of CIN and cancer through the modulation of inflammatory and anti-inflammatory pathways (Curty et al., 2019; He et al., 2024). Moreover, the vaginal microbiota undergoes changes during menopause due to declining estrogen levels, resulting in squamous epithelial thinning, reduction in *Lactobacillus* numbers, increased abundance of anaerobic bacteria, and inflammation (Gorodeski, 2000; Sturdee and Panay, 2010; Sadeghpour Heravi, 2024; Lehtoranta et al., 2022). These alterations contribute to an increased risk of persistent HPV infection and therefore play a crucial role in cervical cancer progression.

Several studies have explored the interplay between the microbiome, immune system, metabolites, cytokines, and microRNAs (miRNAs) in cervical cancer and its precursor lesions (Castanheira et al., 2021; Zhou et al., 2021; Kawahara et al., 2021; Chauhan et al., 2024; Huang et al., 2024). For instance, we had previously identified several diagnostic markers for cervical cancer, including RANTES, miR-20b-5p, miR-155-5p, miR-126-3p, miR-144-3p, miR-451a, and metabolites. These novel biomarkers can detect lesions in cervical mucus with high accuracy, and hold promise for cancer prevention and treatment (Kawasaki et al., 2024; Fujii et al., 2024). However, only a few studies have examined all of these factors using specimens from the same participants. In particular, little is known regarding the differences in cervical and vaginal microbiota between premenopausal and postmenopausal women, and their impact on precursor lesions and invasive cancer. To this end, we performed an integrative analysis of the microbiome, microbial metabolites, cytokines, and miRNAs in premenopausal and postmenopausal women with cervical cancer and its precursor lesions. The objective of this study was to elucidate age-related differences in disease progression and identify potential mechanisms underlying these changes.

## 2 Materials and methods

### 2.1 Patient characteristics and sample collection

Cervicovaginal mucus samples were collected from 296 patients who attended the outpatient clinic at Fujita Health University Hospital, Aichi Prefecture, Japan, for gynecological examinations from July 2017 to January 2021. Patients treated for sexual transmitted diseases including chlamydia, gonorrhea and herpes simplex viruses were excluded. The study protocol was approved by the Ethics Committee of Fujita Health University (HM22-516) and the National Institute of Infectious Diseases. Written informed consent was obtained from each patient. All procedures were performed in accordance with the approved guidelines and regulations.

From each patient, cervicovaginal mucus samples were collected using BD BBL culture swabs (Becton, Dickinson and Company, Franklin Lakes, NJ, USA) for microbiome analysis, separate cotton swabs (1 cm diameter) for metabolomics and miRNA analysis, a cervical brush for HPV genotyping, and a Merocel cervical sponge (Medtronic Xomed, Inc., Jacksonville, FL, USA) for immunoassay. The samples were immediately stored at -20°C in the outpatient ward and then transferred to a -80°C deep freezer.

Based on histology, the samples were categorized as cervical intraepithelial neoplasia (CIN1, n = 17; CIN2, n = 80; CIN3, n = 82) or squamous cell carcinoma (SCC, n = 69; **Table 1** and **Supplementary Table S1**). Only HPV-positive patients were included in the study, and adenocarcinoma cases were excluded. Due to ethical constraints in obtaining cervical biopsies from healthy volunteers, 48 HPV-negative women undergoing infertility treatment were included as normal controls. HPV-negative status was determined by analyzing the exfoliated cervical cells. HPV genotyping was performed using the DNA extracted from cervical brush samples by PCR with PGMY primers, followed by reverse line blot hybridization (Azuma et al., 2014).

**Table 1:** Cervical neoplasia and age distribution.

Histology	Age	Number	Median age
Normal	< 50	48	37
	50-88	0	–
	Total	48	37
CIN1	< 50	17	36.5
	50-88	0	–
	Total	17	36.5
CIN2	< 50	68	37
	50-88	12	55
	Total	80	38
CIN3	< 50	67	35
	50-88	15	59
	Total	82	37
SCC	< 50	26	38
	50-88	43	64
	Total	69	55

Patients in the CIN2, CIN3, and SCC groups were further stratified by age (< 50 years or ≥ 50 years). Analyses were performed using 50 years of age as a threshold, classifying individuals younger than 50 years as premenopausal and those aged 50 years and older as postmenopausal. The exact age at menopause for each individual was not strictly verified, as the timing of menopause varies among individuals and can typically only be determined retrospectively. The median age in the normal, CIN1, CIN2 (< 50 years), CIN2 (50–88 years), CIN3 (< 50 years), CIN3 (50–88 years), SCC (< 50 years), and SCC (50–88 years) groups were 37 years, 36.5 years, 37 years, 55 years, 35 years, 59 years, 38 years, and 64 years respectively.

Due to improper sampling at the time of collection, only 88 samples were available for miRNA analysis (**Supplementary Table S1**).

### 2.2 Library preparation and sequencing for microbial analysis

DNA was extracted from the cervicovaginal mucus samples collected with BD BBL culture swabs using the Charge Switch Forensic DNA Purification Kit (Thermo Fisher Scientific, Waltham, MA, USA), and quantified using a Synergy H1 microplate reader (BioTek, Winooski, VT) and QuantiFluor dsDNA system (Promega, Madison, WI, USA) as per the manufacturers’ instructions. The genomic DNA was amplified by PCR with universal 16S rRNA gene (rDNA) bacterial primers targeting the V3/V4 region, and sequenced using the MiSeq system. To prepare the sequencing library, two PCR amplifications were initially performed with Bakt_341F and Bakt_805R primers, followed by the addition of index primers. The library was quantified as described above, and the quality was assessed using Fragment Analyzer (Advanced Analytical Technologies, Ankeny, IA, USA) and the dsDNA 915 Reagent Kit (Agilent, Santa Clara, CA, USA) according to the manufacturers’ instructions. Paired-end sequencing (2 × 300 bp) was conducted on the Illumina MiSeq platform (Illumina, San Diego, CA, USA) using a MiSeq Reagent Kit v3 (Illumina).

### 2.3 Sequencing data analysis

Reads beginning with sequences that perfectly matched the primer were extracted using the `fastx_barcode_splitter` tool in the FASTX-Toolkit, and the primer sequences were trimmed. The Sickle tool was then employed with a quality threshold of 20 to trim and filter the reads, and the trimmed reads and paired-end reads shorter than 150 bases were removed. The remaining paired-end reads were merged using the FLASH program under the following conditions: merged fragment length of 420 bases, read fragment length of 280 bases, and a minimum overlap length of 10 bases. All merged sequences were retained for further analysis.

Sequence denoising, chimera checking, and taxonomic assignments were performed using QIIME2.0 (2019.4) with default parameters. The Divisive Amplicon Denoising Algorithm 2 (DADA2) method was applied, and taxonomic assignments were made using the Greengenes database (13_8) clustered at 97% identity.

The Ribosomal Database Project (RDP) classifier was utilized for genus-level taxonomic assignments specifically targeting *Lactobacillus*, with merged sequences (reads) serving as input. While the V3–V4 region of the 16S rRNA gene presents limited variability among closely related species, SpeciateIT incorporates additional phylogenetic context to support species-level assignments where possible. Given the limited variability of the 16S rRNA V3–V4 region among closely related species, the SpeciateIT tool was used for species-level classification. To this end, the 16S rRNA gene sequences of 12 *Lactobacillus* species (*L. coleohominis*, *L. crispatus*, *L. gasseri*, *L. iners*, *L. jensenii*, *L. mucosae*, *L. paracasei*, *L. paraplantarum*, *L. plantarum*, *L. reuteri*, *L. rhamnosus*, and *L. vaginalis*)) included in the SpeciateIT database (https://sourceforge.net/projects/speciateit/) were downloaded from the RDP website (http://rdp.cme.msu.edu/hierarchy/hb_intro.jsp) using the following criteria: strain = both; source = isolates; size ≥1200 bases; quality = good; and taxonomy = nomenclatural. Following the SpeciateIT instructions, a database was created from these 16S rRNA gene sequences for species discrimination analysis, and the output sequences were classified as the genus *Lactobacillus* by the RDP classifier.

### 2.4 Metabolite extraction from cervical mucus

To extract metabolites from the cervical mucus samples, each cotton swab was dipped in 500 μL pre-chilled methanol containing a 10 μM internal standard solution (2-morpholinoethanesulfonic acid). Following incubation at 4°C for 30 minutes, 200 μL supernatant was aspirated from each tube and mixed vigorously with 325 μL of pre-chilled chloroform and water (2:1.25, v/v). The samples were centrifuged at 13,500 g for 10 minutes at 4°C, and the supernatants were filtered through a 10 kDa molecular weight cut-off filter (Merck) and then dried under vacuum for metabolomics.

### 2.5 Metabolomics data acquisition

The cervical mucus extracts were diluted with 50 μL water and analyzed using the LCMS-8060 system (Shimadzu Corporation), which features a triple quadrupole mass spectrometer. Liquid chromatography was performed on a Supelco Discovery HS F5–3 column (3 μm, 150 mm × 2 mm; Merck) with mobile phase A (0.1% formic acid in water) and mobile phase B (0.1% formic acid in acetonitrile). The mobile phase flow rate was set to 250 μL/min in gradient mode, and the sample injection volume was 1 μL. The column temperature was maintained at 40°C. The gradient program was as follows: 0% B,0–2 min; 0%–25% B, 2–5 min; 25%–35% B, 5–11 min; 35%–95% B, 11–15 min; and 95% B, 15–20 min. Instrument control was managed using LabSolutions LC-MS software version 5.114 (Shimadzu Co.). Compounds were identified with the LC/MS/MS Method Package for Primary Metabolites version 2 and LabSolutions Insight software version 3.8 SP4 (Shimadzu Co.). Peak areas of each compound were calculated and normalized to the peak area of the internal standard.

### 2.6 Protein extraction from cervical sponges

Protein was extracted from the Merocel cervical sponges as previously described. Each sponge was weighed (wet weight) and placed in a 2-mL Spin-X centrifuge filter tube (Corning, NY, USA) containing 300 μL extraction buffer (phosphate-buffered saline, Sigma-Aldrich, St. Louis, MO, USA), and 256 mM NaCl and 100 μg/mL aprotinin (Wako, Amagasaki, Japan) were added slowly. The sponges were incubated at 4°C for 2 hours, and then centrifuged at 14,000 rpm for 15 minutes at 4°C. The extracts were collected, and 30 μL fetal bovine serum (FBS) was added to 270 μL extract. The samples were briefly vortexed and frozen at −80°C.

### 2.7 Cytometric bead array

RANTES levels were measured by multiplex bead-based immunoassay (Cytometric Bead Array, BD Biosciences, Franklin Lakes, NJ, USA) according to the manufacturer’s protocol. Depending on RANTES levels in the protein extract, thawed cervical mucus extracts were diluted between 1:1 and 1:5 in extraction buffer.

The 10-point standard curve was used to convert the mean fluorescence intensity of each bead cluster into RANTES concentration using FCAP Array™ software (BD version 3.0.1).

RANTES levels in cervical mucus samples were adjusted using the weighted volume method as previously described. To account for variations in sponge weight, a dilution factor was calculated as follows: [(x − y) + 300 mg buffer]/(x − y), where x represents the sponge weight after sample collection and y represents the dry sponge weight. RANTES levels were then multiplied by the dilution factor to obtain weight-normalized values (Marks et al., 2012; Otani et al., 2019).

### 2.8 Real-time RT- PCR

The miRNAs in cervical mucus (hsa-miR-451a, 001141; hsa-miR-20b-5p, 001014; hsa-miR-144-3p, 002676; hsa-miR-126-3p, 002228; hsa-miR-155-5p, 002623; hsa-miR-7109-5p, 466424_mat; hsa-miR-3180, 463043_mat) were quantified by RT-PCR using the TaqMan™ MicroRNA Assay (Thermo Fisher Scientific, Waltham, MA, USA). Briefly, 350 ng total RNA was reverse transcribed using the TaqMan™ MicroRNA Reverse Transcription Kit according to the protocol outlined in the User Bulletin (protocol for ‘Creating Custom RT and Pre-amplification Pools using TaqMan MicroRNA Assays’ #4465407, Thermo Fisher Scientific). Quantitative RT-PCR was performed using TaqMan Array MicroRNA 384-well cards on the 7900 Real-Time PCR system (Thermo Fisher Scientific). The reaction mix included 0.5 μL of 20× TaqMan MicroRNA Assays (including PCR primers and probes with 5′-FAM and 3′-TAMRA), 1 μL of the diluted template, and 5 μL of 2× TaqMan Fast Advanced Master Mix (Thermo Fisher Scientific). The PCR conditions were as follows: initial denaturation at 95°C for 20 seconds, followed by 50 cycles of 95°C for 1 second and 60°C for 20 seconds. Data analysis was performed using RQ Manager 1.2 (Thermo Fisher Scientific), and the fractional cycle number (the cycle at which the amplified target reached a fixed threshold) was determined. MiRNA expression levels were normalized to the average signals of miR-7109-5p and miR-3180, and presented as ΔCt values as previously described. The relative quantity of miRNA in each disease category compared to normal controls was calculated based on the relative ratios between the two conditions (Kotani et al., 2022).

### 2.9 Statistical analysis

The microbiome data was processed, normalized, and profiled using the MicrobiomeAnalyst platform (McGill University, Montreal, Quebec, Canada, https://www.microbiomeanalyst.ca/). A low-count filter was applied to exclude all features with counts below 4, and those present in at least 15% of the samples were retained. Features with less than 10% variance, based on quartile rank, were removed using a low-dispersion filter. The data for all samples were normalized using Total Sum Scaling.

Alpha diversity was calculated using the Chao1, Shannon, and Simpson indices. Beta-diversity analysis was performed to evaluate the differences in microbiome composition between predefined groups using Bray-Curtis distances. The resulting distance matrix was visualized through Principal Coordinates Analysis (PCoA), where each point on the plot represents the overall microbiome composition of a single sample. The statistical significance of the differences in β-diversity was assessed by group centroid positions (PERMANOVA), and the differences in variance between groups (PERMDISP2).

Linear Discriminant Analysis Effect Size (LDA-LefSe) was used to identify taxonomic signatures at the genus level in each group. This method evaluates both statistical significance and biological consistency (effect size). Kruskal-Wallis rank sum test was first performed to identify the significantly different taxa between the groups, followed by LDA to calculate the effect size of each feature. The taxa with *p*-value < 0.05 and LDA score > 3.5 were selected as the candidate markers for distinguishing between the disease and control groups. The microbiome and metabolome data were compared using the Microbial Metabolomics analysis module in MicrobiomeAnalyst, applying MaAsLin2 (Microbiome Multivariable Association with Linear Models (Mallick et al., 2021), and limma (Linear Models for Microarray Data (Ritchie et al., 2015) respectively. The significantly different microbial taxa and metabolites were screened using *p*-value < 0.05 as the cutoff.Spearman’s correlation analysis was performed for these significant features, and the results were visualized in a heatmap based on correlation coefficients.

Data integration analysis was performed using DIABLO (Data Integration Analysis for Biomarker Discovery using Latent Variable approach for Omics studies), a multi-omics integration method that identifies common molecular features across different omics data, with the same working module as MicrobioAnalyst’s correlation analysis. Following feature selection, the microbiota, metabolome, and clinical features with the best discriminative power were identified and retained. The results were presented in a three-dimensional scatter plot.

Univariate and multivariate analyses were performed using MetaboAnalyst 6.0 (https://www.metaboanalyst.ca/). Metabolite intensity values were autoscaled (mean-centered and divided by the SD), and missing values were replaced with 20% of the minimum positive value. Volcano plot analysis was performed on the normalized data to identify significant metabolic changes using −log10(p) >1.3 (p < 0.05) and |log2(FC)| >1 (FC > 2) as the thresholds.

The first filter in the hierarchical heatmap of the microbiome and metabolites was divided into the respective “Low” and “High” categories for miR-451a, miR-20b-5p, miR-144-3p, miR-126-3p, miR-155-5p, and RANTTES based on the cutoff values presented in our previous paper (Fujii et al., 2024).

## 3 Results

### 3.1 Cervical neoplasia is associated with an altered cervicovaginal microbiota

**Figure 1:**
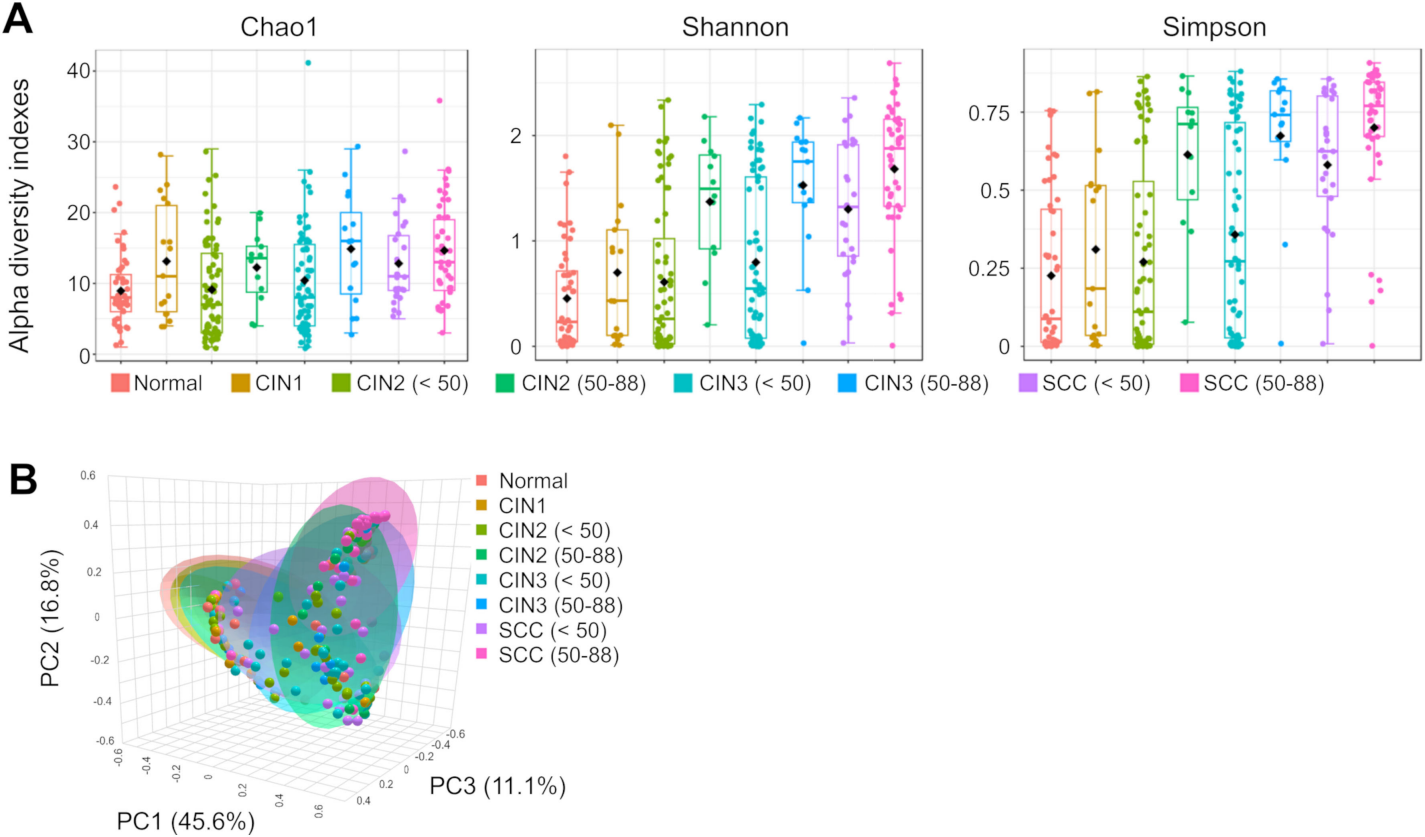
Analysis of the diversity of cervical microbiota. **(A)** The Chao1, Shannon and Simpson α-diversity indices were used to estimate microbial diversity for disease group, and data was compared using Wilcoxon rank-sum test and Kruskal-Wallis test. **(B)** Principal coordinates analysis (PCoA) corresponding to the Bray–Curtis dissimilarity index (β-diversity). Each dot represents a sample subject. PERMANOVA results indicated a significant difference (*p*-value: 0.001), and PERMDISP analysis showed dispersion effects (*p*-value: 0.0033266). The results of the significant tests between two groups are shown in **Supplementary Table S2**.

A total of 10,114,357 reads (average 34,170.1 reads per sample) and 357 OTUs (average 13 OTUs per sample) were obtained from 296 cervicovaginal mucus samples. We analyzed the α-diversity of the microbiota at the genus level for each group, and then evaluated species richness and evenness using the Chao1, Shannon, and Simpson indices (**Figure 1A**; **Supplementary Table S2**). Regardless of age, significant differences in microbial diversity were evident between the normal and SCC groups (*p* < 0.01). Furthermore, the α-diversity differed significantly between the CIN3 and SCC groups among those under 50 years of age (*p* < 0.01). In the SCC group, α-diversity differed significantly between those under 50 years of age and those aged 50 years and over (*p* < 0.01).

The similarity of the cervical microbiota between the groups was evaluated using β-diversity. Compared to the normal group, species similarity was significantly lower in the CIN3 or worse groups among those under 50 years of age (pairwise PERMANOVA: *p* < 0.01). In those aged 50 years and over, this trend was observed in the CIN2 or worse groups (PERMANOVA: *p* < 0.01, PERMDISP: *p* < 0.01, **Figure 1B**; **Supplementary Table S2**). There was also a significant difference in microbial community similarity between those under and over 50 years of age in the CIN2, CIN3, and SCC groups (*p* < 0.01).

### 3.2 Neoplasia-associated changes in the cervicovaginal microbiota are influenced by age

**Figure 2:**
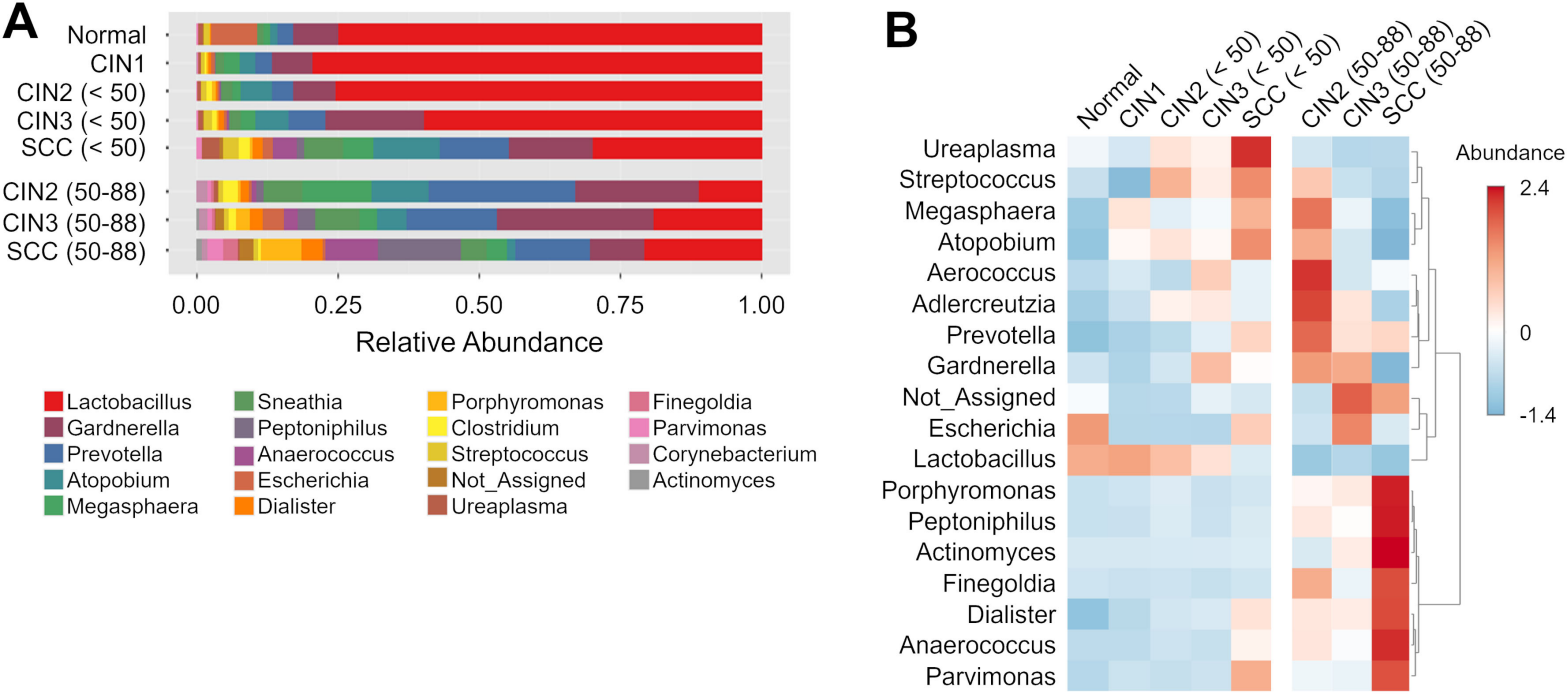
Taxonomic composition and abundance of the cervical microbiome **(A)** The relative abundance of different genera in each group. **(B)** Heatmap showing the hierarchical clustering of samples based on the relative abundance of the top 18 genera in cervical neoplasia groups.

**Figure 3:**
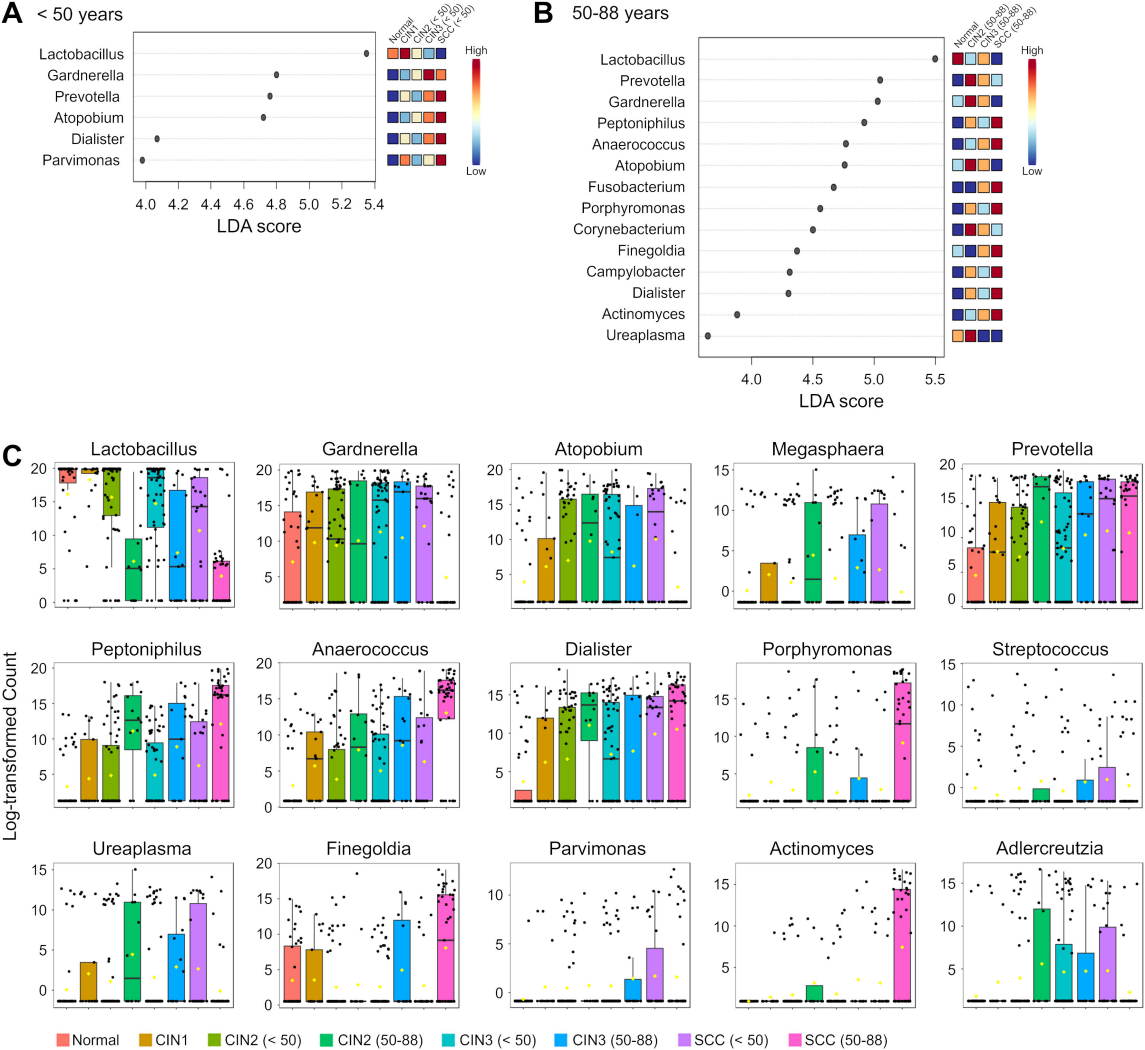
Differential abundance of genera in the cervicovaginal microbiota across disease groups and age groups. **(A, B)** Linear discriminant analysis (LDA) scores of differentially abundant genera in those aged **(A)** < 50 years and **(B)** 50–88 years in the indicated groups. Genera with Kruskal-Wallis p-value < 0.05 and LDA score > 3.5 are shown. The colors represent the relative abundance of these specific genera in each disease group. **(C)** Box plots showing bacterial abundance at the genus level across disease groups. The data are presented as log-transformed counts.

The relative abundance of different microbial genera was compared between the disease groups, with each stratified by those under and over 50 years of age (**Figures 2**, **3**). The dominant bacterial genera showed age-related differences in the CIN2, CIN3, and SCC groups. *Atopobium*, *Streptococcus*, and *Ureaplasma* were more prevalent in those under 50 years of age with CIN2, CIN3 and SCC compared to the age-matched normal controls, whereas *Peptoniphilus*, *Porphyromonas*, *Finegoldia*, and *Actinomyces* were predominantly observed in those aged 50 years and over. A decrease in *Lactobacillus* abundance was observed with disease progression in those under 50 years of age. In contrast, a significant reduction in *Lactobacillus* was already evident at CIN2 among those aged 50 years and over, with this proportion remaining nearly unchanged through to SCC.

LEfSe analysis was performed to identify the candidate genera of each group, and several taxa with discriminatory ability were detected in the disease groups compared to the normal group (**Figure 3A**). *Dialister* was significantly more abundant in the SCC group compared to the normal group irrespective of age, indicating its diagnostic potential for SCC across all ages. Furthermore, *Prevotella* and *Atopobium* demonstrated high discriminatory ability in those under 50 years of age with SCC but not for those aged 50 years and over (LDA score >4.5). Conversely, *Peptoniphilus*, *Anaerococcus*, *Fusobacterium* and *Porphyromonas* could only distinguish in those aged 50 years and over with the SCC group from the normal controls.

**Figure 4:**
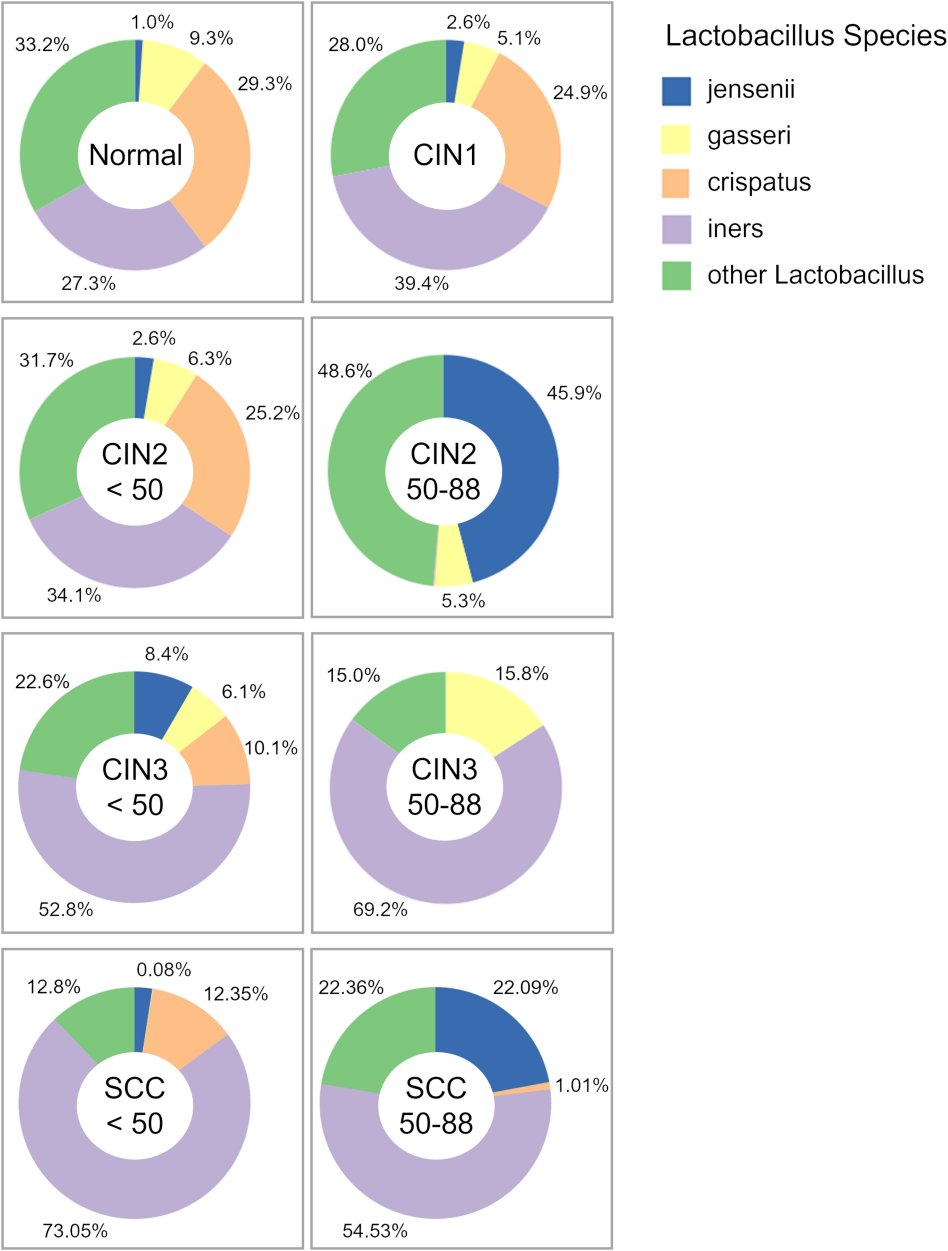
Pie charts of the relative abundance of *Lactobacillus* species. Major bacterial species are shown in the key, while species that do not meet the data filtering criteria – due to low abundance or insufficient detection across specimens – have been grouped under “other *Lactobacillus*.”.

We also analyzed the distribution of *Lactobacillus* species, and found that the prevalence of *L. iners* increased with disease progression among those under 50 years of age, and was highest in the SCC group. As shown in **Figure 4**, the prevalence of *L. iners* in the normal, in those under 50 years of age with SCC were 27.3%, 73%, andrespectively. In addition, *L. crispatus* showed a decrease in those under 50 years of age from the CIN1 group, and was notably reduced in the CIN3 and SCC groups. On the other hand, we did not observe any consistent trends for *Lactobacillus* species in those aged 50 years and over. *L. gasseri* was a dominant species in the normal and CIN groups regardless of age, but its abundance decreased to less than 0.1% in the SCC group. Taken together, the cervicovaginal microbiota exhibits disease-specific characteristics that are significantly dependent on age.

### 3.3 The cervicovaginal microbiome correlates with the metabolome in cervical neoplasia

**Figure 5:**
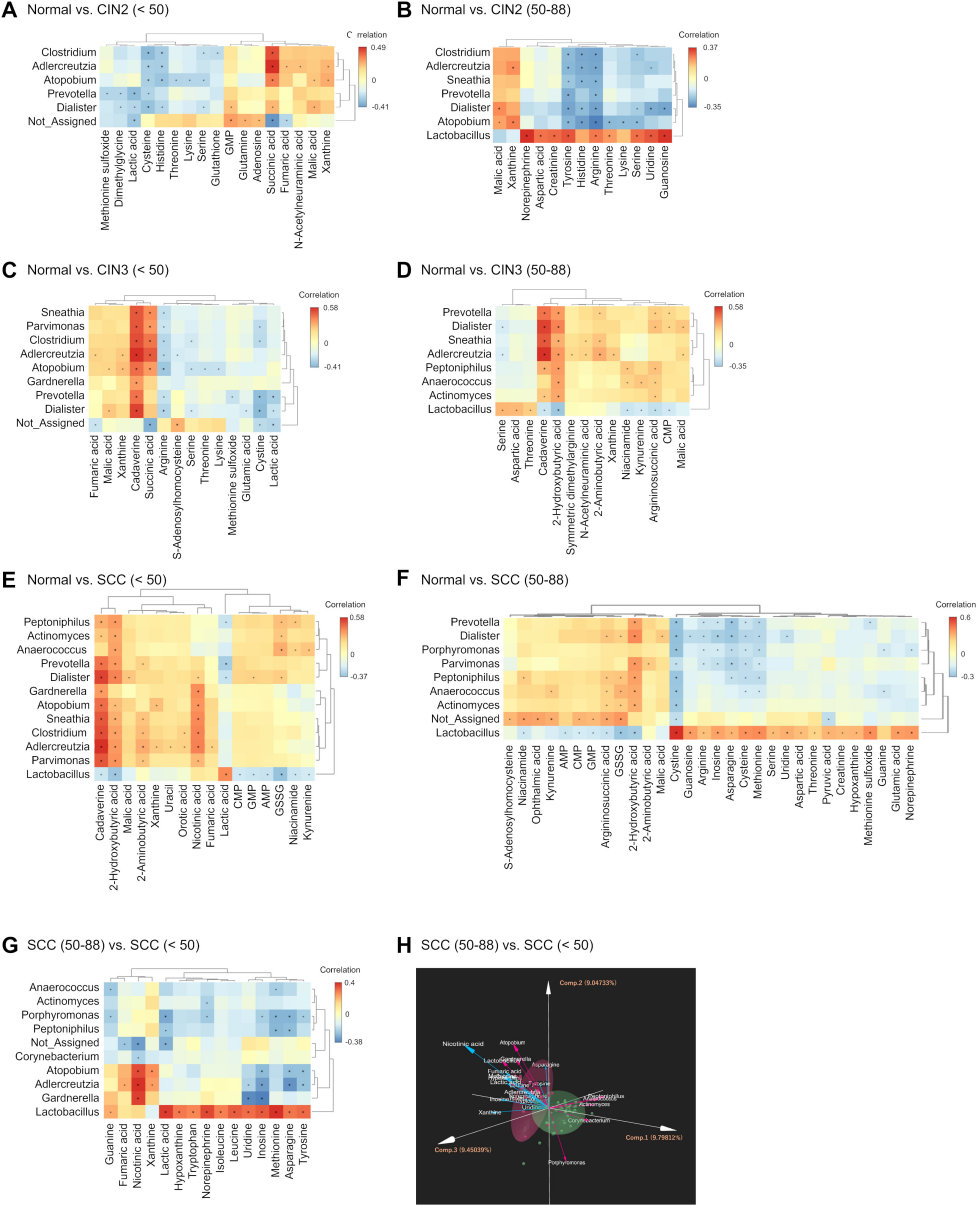
Integrated correlation analysis of microbiota and metabolites. **(A–G)** Heatmaps depicting the correlation and hierarchical clustering of differential microbiota and metabolites in **(A)** CIN2 (< 50 years), **(B)** CIN2 (50–88 years), **(C)** CIN3 (< 50 years), **(D)** CIN3 (50–88 years), **(E)** SCC (< 50 years), and **(F)** SCC (50–88 years) groups compared to the normal group, and in **(G)** SCC (< 50 years) group compared to SCC (50–88 years) group. Red indicates positive correlation and blue indicates negative correlation. Significant correlations are indicated by *p < 0.05. **(H)** Comparison of the microbiome and metabolome data between SCC (< 50 years) and SCC (50–88 years) groups using DIABLO. The percentage values in parentheses next to PC1, PC2, and PC3 represent the variance explained by each component. The length of the vectors indicates the strength of influence of each genus or metabolite. Each data point represents an individual patient color-coded by group.

The association between the cervicovaginal microbiota and metabolites was evaluated by Spearman correlation analysis (**Figure 5**; **Supplementary Table S3**). The correlation between microbial taxa and metabolites that showed significant differences between the neoplasia and normal groups were analyzed, followed by microbiota-metabolite correlations between age groups within each disease category. Among those under 50 years of age, five common microbiota-metabolite correlations were identified in the CIN2, CIN3 and SCC groups compared to the normal group. *Adlercreutzia* was positively correlated with fumaric acid and xanthine, and *Atopobium* with xanthine (*p* < 0.0001), while *Prevotella* and *Dialister* correlated negatively with lactic acid (**Figures 5A, C, E**). Furthermore, *Adlercreutzia*, *Dialister*, *Clostridium*, *Parvimonas*, *Sneathia*, *Atopobium*, *Prevotella*, and *Gardnerella* showed consistent positive correlations with cadaverine among those under 50 years of age in the CIN3 and SCC groups. In those under 50 years of age with SCC, *Atopobium* and *Sneathia* correlated positively with cadaverine, 2-hydroxybutyric acid, and nicotinic acid (*p* < 0.0001; **Figure 5E**). Among those aged 50 years and over, consistent correlations were observed between 2-hydroxybutyric acid and several taxa in the CIN3 and SCC groups (**Figures 5B, D, F**), while *Peptoniphilus* correlated positively with argininosuccinic acid, and negatively with cystine in the SCC group (**Figure 5F**). In addition, significant positive correlations were observed between *Atopobium*, *Adlercreutzia*, *Gardnerella*, and nicotinic acid in those under 50 years of age compared to those aged 50 years and over, and similar trends were confirmed in the DIABLO analysis (**Figures 5G, H**). Age-dependent correlations between microbiota and metabolites were also observed in the CIN2 and CIN3 groups (**Supplementary Figure S1**). Taken together, correlations between the cervicovaginalmicrobiota and metabolites were consistent in those under 50 years of age with cervical neoplasia, but infrequent in those aged 50 years and over. Moreover, these correlations differed significantly between age groups within the same disease category.

### 3.4 The dominant microbial taxa in SCC correlates with RANTES and miRNAs

**Figure 6:**
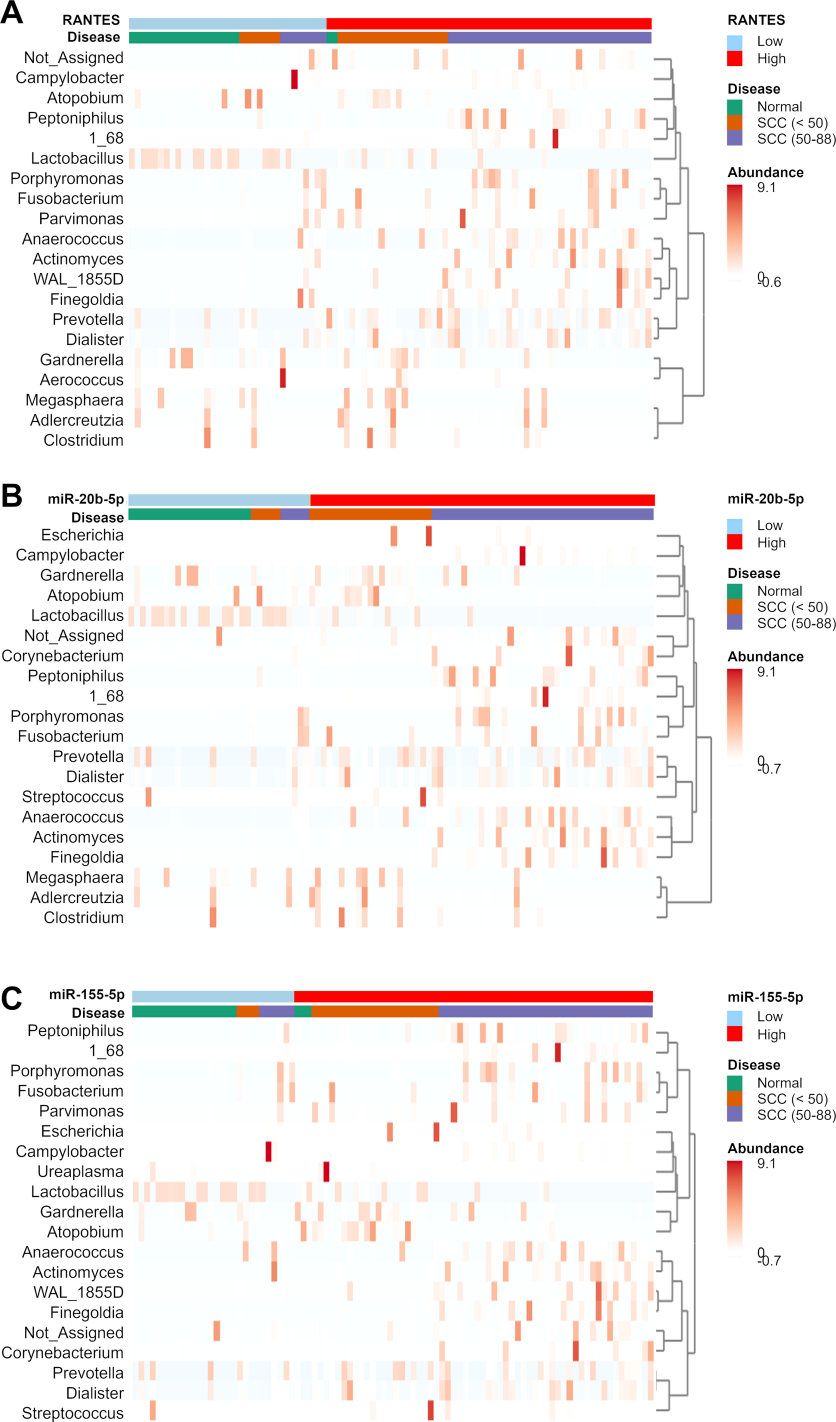
Clustering heatmap showing the relative abundance of bacterial genera in the disease groups. The disease groups are stratified by the expression levels of **(A)** RANTES, **(B)** miR-20b-5p, and **(C)** miR-155-5p. The cutoff values have been mentioned in our previous study (Fujii et al., 2024). Bacterial genera that showed significant differences between the disease and normal groups were used in the clustering analysis.

We analyzed the relationship between the cervicovaginal microbiome and the expression levels of RANTES, miR-20b-5p, miR-155-5p, miR-126-3p, miR-144-3p, and miR-451a. RANTES, miR-20b-5p, and miR-155-5p showed significant correlation with multiple bacterial genera (**Figure 6**; **Supplementary Figure S2**). In the SCC group, the relative abundance of *Prevotella* increased in the high levels of RANTES, miR-20b-5p, and miR-155-5p subgroups regardless of age, while *Gardnerella* and *Atopobium* increased in those under 50 years of age, and the abundance of *Peptonophilus*, *Anaerococcus*, and *Finegoldia* were higher in those aged 50 years and over (**Figures 6A–C**).

### 3.5 Association between RANTES, miRNAs, and metabolites in cervical SCC

**Figure 7:**
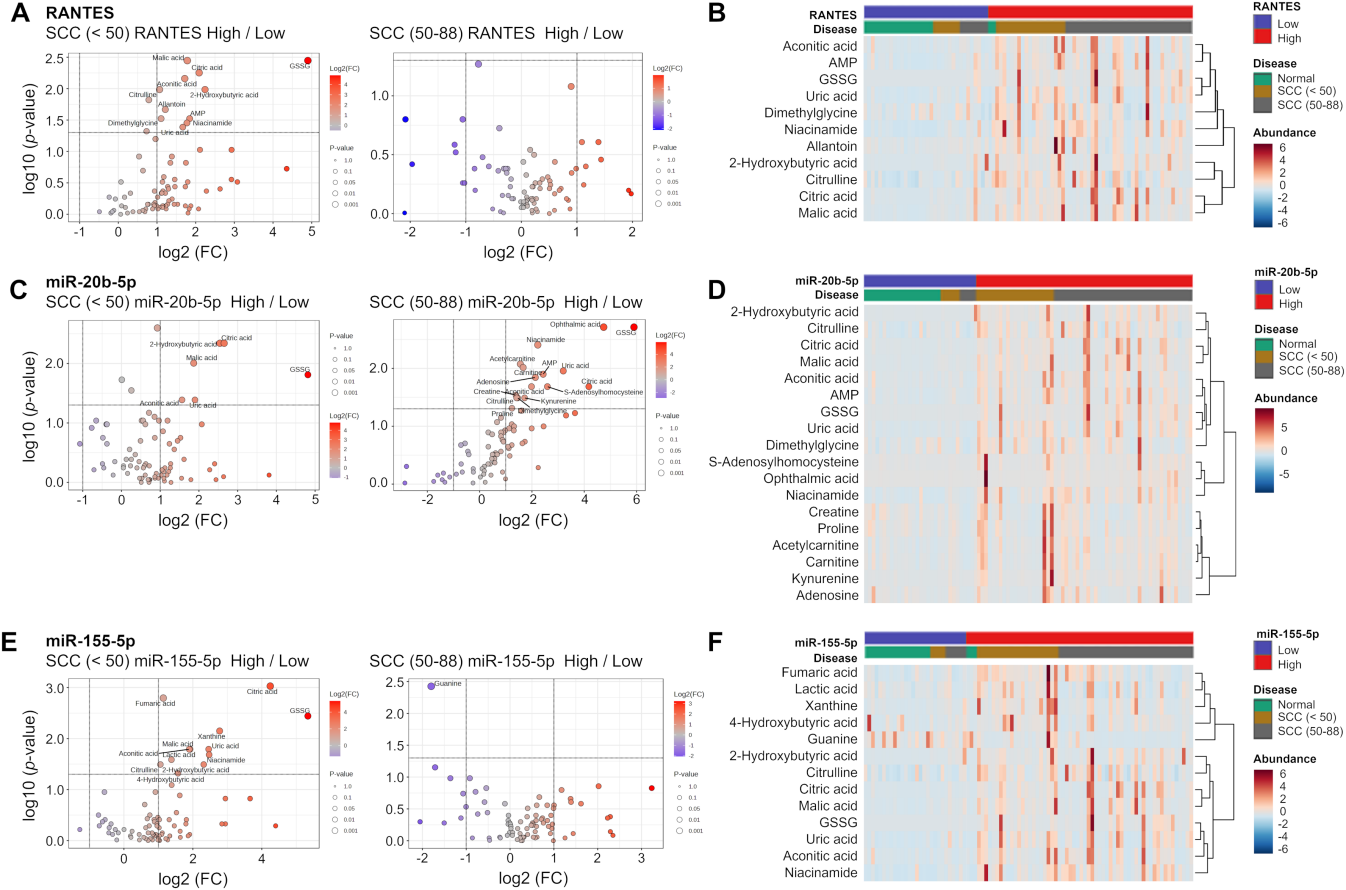
Volcano plot analysis and clustering heatmap of RANTES, miR-20b-5p and miR-155-5p. **(A, C, E)** Volcano plots showing distribution of metabolites in the SCC (<50) and SCC (50-88) groups stratified respectively by the expression levels of RANTES, miR-20b-5p and miR-155-5p. The volcano plot displays −log10(P-value) on the y-axis and log2(fold change) on the x-axis. Each point represents a metabolite, the circle size indicates the *p*-value, and stronger color intensity indicates higher log2(fold change). **(B, D, F)** Heatmaps showing the relative changes in metabolites identified in the Volcano plot analysis. The color ranges from deep red (indicating high abundance or level) to deep blue (indicating low abundance or level), and white indicates no change.

The expression levels of RANTES, miR-20b-5p, and miR-155-5p were also associated with specific metabolites in the SCC group. As shown in **Figure 7**, GSSG, malic acid, citric acid, aconitic acid, 2-hydroxybutyric acid, and uric acid were consistently elevated in those under 50 years of age with higher levels of RANTES, miR-20b-5p, and miR-155-5p. In contrast, no significant differences in these metabolites were observed in those aged 50 years and over. Taken together, several metabolites were significantly increased in SCC with the high levels of RANTES, miR-20b-5p, and miR-155-5p, and these changes were more pronounced in those under 50 years of age.

## 4 Discussion

We identified age-specific differences in the cervical microbiome of premenopausal and postmenopausal women with cervical neoplasia, which were associated with metabolites, RANTES, and miRNAs (miR-155-5p, miR-20b-5p). These findings suggest that the role of the cervical microbiome in cervical neoplasia depends on age, and that these menopause-associated changes may influence neoplasia initiation and progression.

Previous studies have reported that changes in the cervicovaginal microbiota can serve as potential biomarkers for HPV infection and cervical cancer (Fong Amaris et al., 2024; Dong et al., 2022; Han et al., 2023; Kang et al., 2021; So et al., 2020; Wu et al., 2021; Głowienka-Stodolak et al., 2024). For instance, *Atopobium vaginae* is associated with an increased risk of high grade CINs in HPV-infected individuals (Mitra et al., 2016). In line with these studies, we also identified distinct microbiota characteristics in each disease group. Notably, *Atopobium* and *Prevotella* can distinguish pre-menopausal SCC women from age-matched healthy controls, while *Porphyromonas*, *Peptoniphilus*, *Anaerococcus* and *Fusobacterium* may serve as useful diagnostic markers for SCC among elderly women. Our study is the first to demonstrate that distinct microbial taxa could serve as markers for SCC before and after menopause. Furthermore, the abundance of *Lactobacillus* species also showed age-related differences. In those under 50 years of age, *L. iners* became the dominant species with the progression of cervical lesions, while the abundance of *L. crispatus* decreased. The vaginal microbiome undergoes significant changes after menopause, typically exhibiting increased diversity and lower abundance of the *Lactobacillus* genus (Vargas-Robles et al., 2023; Li et al., 2022). Similar changes have been reported with the progression of CIN as well (Audirac-Chalifour et al., 2016; Fong Amaris et al., 2024; Teka et al., 2023). *L. crispatus* and *L. gasseri* produce both D-lactic acid and L-lactic acid, whereas *L. iners* primarily produces L-lactic acid (Witkin et al., 2013). Deficiency in D-lactic acid and increased levels of L-lactic acid may lower DNA repair capacity, potentially contributing to cancer progression (Latham et al., 2012; Wagner et al., 2015). These findings suggest that age-related changes in the cervicovaginal microbiota may serve as disease indicators, and shed light on the mechanisms by which microbiota-derived metabolites and immune regulation contribute to SCC progression.

A consistent correlation between the microbiome and metabolome was observed in those under 50 years of age with CIN2 or worse, but not in those aged 50 years and over. For instance, *Atopobium* showed a positive correlation with xanthine, an inflammation-related metabolite (Farr Zuend et al., 2023), in those under 50 years of age. We had previously identifies xanthine as a promising biomarker for CINs and SCC (Kawasaki et al., 2024), suggesting a possible link between *Atopobium* and xanthine metabolism in younger women. Our results also revealed age-specific differences in microbiota-metabolite correlations. The positive association between *Atopobium* and xanthine observed in younger women with cervical neoplasia may indicate a microbial contribution to an inflammatory cervical environment. Xanthine is a purine degradation product that can serve as a substrate for xanthine oxidase, an enzyme known to produce reactive oxygen species (ROS) and promote inflammatory cytokine secretion. Previous studies have shown that xanthine oxidase activity contributes to IL-1β production via inflammasome activation in macrophages (Ives et al., 2015). Our previous study (Kawahara et al., 2021) demonstrated that *Atopobium vaginae* was linked to increased cervical levels of pro-inflammatory cytokines such as IL-1β and TNF-α in patients with CIN, and that its abundance declined after surgical treatment. These findings raise the possibility that *Atopobium* may help sustain an inflammatory microenvironment, potentially through metabolic pathways involving xanthine metabolism, although further functional studies are needed to clarify the underlying mechanisms.

Cadaverine, a metabolite produced by anaerobic bacteria (Ma et al., 2017), showed a positive correlation with eight bacterial species, including *Atopobium*, in those under 50 years of age with the CIN3 and SCC groups. In those aged 50 years and over, however, several bacterial species were positively correlated with 2-hydroxybutyric acid, a metabolite associated with gut bacteria (Qin et al., 2023). Furthermore, *Atopobium*, *Adlercreutzia*, and *Gardnerella* showed a positive correlation with nicotinic acid, a NAD^+^ coenzyme precursor that plays a role in immune cell activation (Yoshii et al., 2019; Chellappa et al., 2022), in those under 50 years of age with the SCC group. A recent study showed that *Gardnerella vaginalis* may enhance the survival of *Atopobium vaginae*, and therefore influence pathogenicity (Castro et al., 2020). Further studies are needed to clarify how these bacterial interactions influence nicotinic acid metabolism.

Overall, these findings highlight age-related differences in the microbiome and metabolome associated with high-grade CIN and cervical cancer, suggesting distinct mechanisms of disease progression. In younger women, the precancerous lesions and invasive cancer have similar microbiota, indicating that the conditions leading to cancer development may already be established in precancerous lesions. On the other hand, the absence of a consistent correlation between the microbiota and metabolites in those aged 50 years and over suggests different mechanisms of disease onset and progression. Understanding these interactions is crucial for elucidating the age-related development of cervical cancer, and identifying potential biomarkers for early detection and intervention.

The expression levels of RANTES, miR-20b-5p, miR-126-3p, and miR-155-5p were associated with specific microbial taxa and metabolites, with notable age-related differences. Previous studies have reported that the levels of various cytokines and chemokines are elevated during the progression of cervical lesions (Kalkusova et al., 2022; Kyrgiou and Moscicki, 2022). RANTES, a chemokine crucial for T-cell immune responses and antiviral defense (Crawford et al., 2011), is induced by *Atopobium vaginae* infection *in vitro*, and promotes inflammation in cervical cells (Łaniewski et al., 2020).

Furthermore, miR-155-5p can promote inflammation and immune cell activation via the NF-κB pathway, and contribute to tumorigenesis (Ma et al., 2011). In the context of HPV infection, elevated miR-155-5p may support persistent inflammation and immune dysregulation, which are key factors in cervical carcinogenesis (Kalkusova et al., 2022, Park et al., 2017). MiR-20b-5p interacts directly with PD-L1 and fosters immune evasion through the PD-L1/PD-1 axis, which is a critical mechanism in cervical cancer progression (Jiang and Zou, 2022). Elevated levels of both miR-155-5p and miR-20b-5p in younger SCC patients may thus contribute to immune dysregulation and tumor progression, supporting the link between persistent HPV infection and the development of cervical neoplasia.

In the SCC group, *Gardnerella* and *Atopobium* were the predominant genera in those under 50 years of age with the high levels of RANTES, miR-20b-5p, and miR-155-5p, and less prevalent in those aged 50 years and over. Taken together, the interactions among microbiota, miRNAs, and cytokines are more pronounced in younger women than in elderly women, and may underlie distinct mechanisms of cervical cancer development and progression. In particular, *Atopobium* emerged as a key genus in multiple analyses, highlighting its strong influence on disease progression in younger women. These variations in the microbiome may be attributed to age-specific differences in estrogen levels. Classifying disease groups by menopausal status and integrating microbiome, metabolome, cytokine, and miRNA analyses could offer valuable insights for age-stratified personalized therapies. Further research incorporating HPV types and other risk factors will be essential to refine these findings and advance personalized medicine approaches for cervical cancer.

This study has several limitations that ought to be considered. One major limitation of this study is its cross-sectional design, which restricts our ability to draw causal inferences regarding the associations between the cervical microbiome, metabolome, immune responses, and lesion severity. It remains unclear whether the observed microbial and metabolic alterations precede lesion development, occur concurrently, or develop as a result of disease progression. Longitudinal cohort studies tracking individual patients over time are needed to determine the temporal dynamics of these factors and to assess their potential as early predictive biomarkers for cervical lesion progression. Additionally, the direct causal relationships between the microbiome, metabolome, cytokines, and miRNAs were not verified, and will require further *in vitro* experiments. While this study aimed to identify associations between the cervicovaginal microbiota and cervical neoplasia in relation to age and HPV status, the causal direction of microbiome alterations remains unclear. It is yet to be determined whether HPV infection leads to changes in the local microbial community or whether such changes precede and potentially influence disease progression. Previous studies (Oh et al., 2015, Shannon et al., 2017) have proposed potential mechanisms that can guide future investigations. Also, we focused exclusively on HPV-positive cervical neoplasia, primarily involving HPV16 and 18, which limits insights into other HPV types and HPV-negative cases.

We also cannot exclude the possibility that age and clinical stage may have acted as confounding factors, especially given the small number of elderly women in the normal and CIN1 groups. Although we stratified the participants by mean menopausal age, it is important to acknowledge that aging is associated with multiple physiological changes, such as immunosenescence, hormonal decline, and increased prevalence of diabetes or cardiovascular disease, that may influence the vaginal microbiome independent of menopause. Future studies incorporating multivariate analyses that adjust for these age-related confounders would further clarify their relative contributions. In addition, we did not collect data on potential confounding factors such as antibiotic or probiotic use, sexual activity, or hormonal contraceptive use. Sampling was performed outside of the menstrual period, but not standardized to a specific phase of the cycle. Furthermore, patients undergoing treatment for sexually transmitted infections were excluded, and subclinical infections and smoking status were also not analyzed. These factors are known to influence the cervicovaginal microbiome and immune environment, and should be considered in future studies to better understand the observed associations.

Although 16S rRNA gene sequencing is widely used for taxonomic profiling, it lacks the resolution to distinguish microbial strains and does not capture functional gene content. Future studies utilizing shotgun metagenomic sequencing may provide deeper insights into microbial species and strain diversity, the functional pathways influencing host immune responses (e.g., RANTES or miRNA expression), and HPV genotyping or integration events (Franzosa et al., 2018). Another limitation of this study concerns the variability in sampling methods. Based on our previous experience, we selected the most appropriate methods, i.e., swab, brush, or sponge, depending on the specific molecular targets of interest. Since it is not feasible to accurately analyze all target molecules from a single specimen type, the choice of sampling tool was optimized for each assay. However, differences in sampling methods may influence the results, and we did not perform a comparative analysis of sampling techniques. Future studies should consider standardizing or evaluating sampling methods to better understand their impact on molecular profiling. Lastly, our metabolome analysis did not include lipid data, which could be relevant to inflammation and cervical neoplasia progression, and will have to be explored in future studies.

Despite these limitations, this study provides novel insights into the molecular networks involved in the onset and progression of premenopausal and postmenopausal cervical neoplasia. The age-specific differences in the cervicovaginal microbiome likely contributes to distinct mechanisms of cervical cancer development and progression by influencing molecular factors such as RANTES, miR-20b-5p, and miR-155-5p. Our findings offer an integrated perspective of cervical neoplasia that may aid in developing age-stratified diagnostic and therapeutic strategies. Further studies considering HPV types and other risk factors are needed to bolster the current results and improve personalized therapies.

## Data Availability

The original contributions presented in the study are publicly available. This data can be found here: https://www.ebi.ac.uk/metabolights/MTBLS12691.
