# Supplementary material for: Distinct cervical microbiome and metabolite profiles before and after menopause: implications for cervical cancer progression
Source: Front Cell Infect Microbiol. 2025 Jul 16;15:1589277. doi: 10.3389/fcimb.2025.1589277 (PMC12307382; doi:10.3389/fcimb.2025.1589277)
Supplement: Supplementary file 1 [file DataSheet1.docx]

Supplementary Material


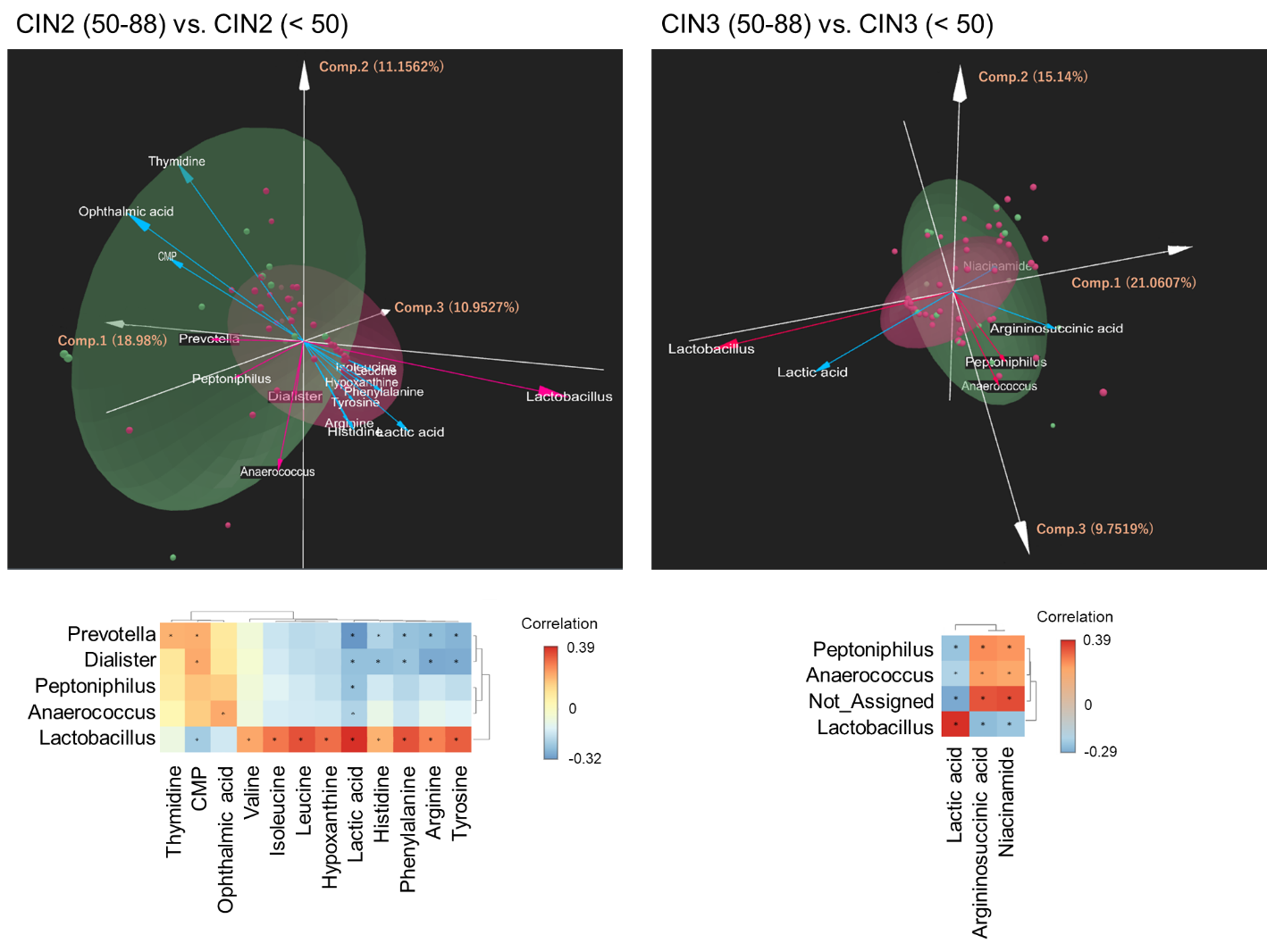


**Figure S1.** Comparison of the microbiome and metabolome data between the younger (<50 years) and elderly (50-88 years) age groups in the CIN2 and CIN3 groups using Principal Component Analysis (PCA). The percentage values in parentheses next to PC1, PC2, and PC3 represent the variance explained by each component. The length of the vectors indicates the strength of influence of each genus or metabolite. Each data point represents an individual patient color-coded by group.
