# Supplementary material for: Distinct cervical microbiome and metabolite profiles before and after menopause: implications for cervical cancer progression
Source: Front Cell Infect Microbiol. 2025 Jul 16;15:1589277. doi: 10.3389/fcimb.2025.1589277 (PMC12307382; doi:10.3389/fcimb.2025.1589277)
Supplement: Supplementary file 2 [file DataSheet2.docx]

Supplementary Material


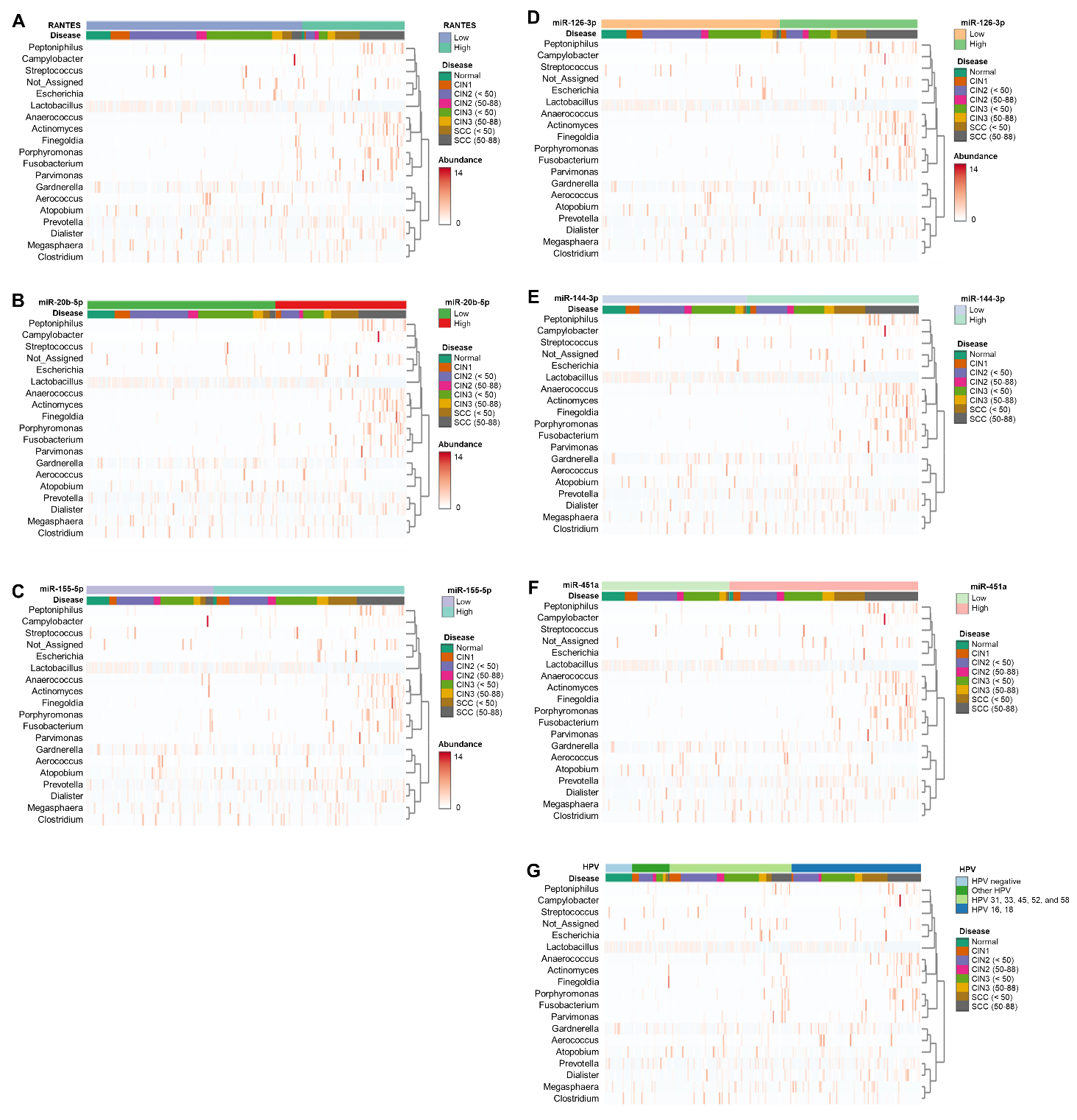


**Figure S2.** Clustering heatmap showing the relative abundance of bacterial genera in the disease groups categorized by (A) RANTES, (B) miR-20b-5p, and (C) miR-155-5p, (D) miR-126-3p, (E) miR-144-3p, (F) miR-451a and (G) HPV status. The miRNAs were classified based on the cutoff values presented in our previous study. Bacterial genera that showed significant differences between the disease and normal groups were used in the clustering analysis.
